# Cardiac energetics in patients with chronic heart failure and iron deficiency: an *in‐vivo* ^31^P magnetic resonance spectroscopy study

**DOI:** 10.1002/ejhf.2454

**Published:** 2022-03-08

**Authors:** Francesco Papalia, Fadi Jouhra, George Amin‐Youssef, Ajay M. Shah, Geoffrey Charles‐Edwards, Darlington O. Okonko

**Affiliations:** ^1^ King's College London British Heart Foundation Centre of Excellence School of Cardiovascular Medicine and Sciences, James Black Centre London UK; ^2^ Guy's and St Thomas' NHS Foundation Trust London UK; ^3^ Lewisham & Greenwich NHS Foundation Trust London UK; ^4^ St George's University Hospitals NHS Foundation Trust London UK; ^5^ King's College Hospital NHS Foundation Trust London UK; ^6^ School of Imaging Sciences and Biomedical Engineering King's College London UK

**Keywords:** Heart failure, Iron, Cardiac energetics, Spectroscopy

## Abstract

**Aims:**

Iron deficiency (ID) is prevalent and adverse in chronic heart failure (CHF) but few human studies have explored the myocardial mechanism(s) that potentially underlie this adversity. Because mitochondrial oxidative phosphorylation (OXPHOS) provides over 90% of the hearts adenosine triphosphate (ATP), and iron is critical for OXPHOS, we hypothesized that patients with CHF and ID would harbour greater cardiac energetic impairments than patients without ID.

**Methods and results:**

Phosphorus magnetic resonance spectroscopy was used to quantify the phosphocreatine (PCr) to ATP (PCr/ATP) ratio, an index of *in‐vivo* cardiac energetics, in CHF patients and healthy volunteers. Cardiac structure and function was assessed from magnetic resonance short stack cines. Patients with (*n* = 27) and without (*n* = 12) ID, and healthy volunteers (*n* = 11), were similar with respect to age and gender. The PCr/ATP ratio was lower in patients with ID (1.03 [0.83–1.38]) compared to those without ID (1.72 [1.51–2.26], *p* < 0.01) and healthy volunteers (1.39 [1.10–3.68], *p* < 0.05). This was despite no difference in cardiac structure and function between patients with and without ID, and despite adjustment for the presence of anaemia, haemoglobin levels, cardiac rhythm, or New York Heart Association (NYHA) class. In the total CHF cohort, the PCr/ATP ratio correlated with ferritin levels (rho = 0.4, *p* < 0.01), and was higher in NYHA class I than class II or III patients (*p* = 0.02).

**Conclusion:**

Iron deficiency is associated with greater cardiac energetic impairment in patients with CHF irrespective of anaemia and cardiac structure and function. Suppression of cardiac mitochondrial function might therefore be a mechanism via which ID worsens human CHF.

## Introduction

Iron deficiency (ID) is prevalent and adverse in chronic heart failure (CHF),^1^, ^2^ but few human studies have explored the myocardial mechanism(s) that potentially drive this adversity. In a prior analysis, iron repletion with iron isomaltoside augmented skeletal muscle energetics in patients with CHF.^3^ This implies that ID impairs skeletal muscle mitochondrial function in CHF patients, and might be similarly injurious to their failing myocardium.

The heart requires a continuous supply of adenosine triphosphate (ATP) to power contractile function and early diastolic relaxation, and to maintain ion channels that orchestrate normal sinus impulse propagation. Mitochondrial oxidative phosphorylation (OXPHOS) provides over 90% of this ATP by coupling fuel substrate oxidation (via glycolysis, fatty acid β‐oxidation, and the Krebs cycle) to ATP synthesis via the electron transport chain (ETC).^4^ Phosphocreatine (PCr), the primary energy buffer in the heart, acts to carry the high‐energy phosphoryl group from ATP to the cytosol where ATP is regenerated via the creatine kinase reaction to fuel activity.^5^ Because PCr consumption maintains ATP levels when ATP demand outstrips supply, the PCr/ATP ratio is a marker of cellular energetic status that is diminished when ATP demand is excessive or when ATP supply is attenuated.

Iron is theoretically indispensable for mitochondrial ATP generation as iron‐containing prosthetic groups (haem and iron–sulfur clusters) are functional components of haemoglobin (Hb) and myoglobin, and of enzymes of the Krebs cycle, β‐oxidation pathway, and the ETC.^6^, ^7^, ^8^, ^9^ Iron is also embedded in catalase and upregulates other antioxidants to maintain a permissive redox environment for OXPHOS.^10^ In *in‐vitro* and pre‐clinical animal models, cellular iron deprivation downregulates Krebs cycle, antioxidant and ETC proteins, blunts mitochondrial respiration (a proxy for cellular energetics), and, when severe, induces lethal murine cardiomyopathy even in the absence of anaemia.^11^, ^12^, ^13^, ^14^ In contrast, in the only prior study to assess cardiac energetics in CHF patients with ID, mitochondrial respiration was preserved despite lower antioxidant and Krebs cycle enzyme levels in heart biopsies.^15^ Because mitochondria were assessed *ex‐vivo* using cardiac homogenates in this study, it is still unclear whether ID is linked to lower myocardial energetics in CHF patients.

Phosphorus magnetic resonance spectroscopy (^31^P‐MRS) is the gold‐standard means of quantifying cardiac energetics *in vivo* and has revealed low PCr/ATP ratios in CHF.^16^, ^17^ Using this technique, we tested the hypothesis that cardiac energetics would be diminished in CHF patients with ID compared to those without, and that diminutions would be independent of Hb and of cardiac morphology and function.

## Methods

### Study design and patients

This was a prospective observational cohort study involving a screening visit and a single ^31^P‐MRS study visit. Consecutively eligible patients were recruited from four dedicated CHF clinics. Eligibility criteria were age 18 to 89 years, left ventricular ejection fraction (LVEF) <45% within the last 6 months, non‐ischaemic cardiomyopathy, stable CHF for 4 weeks with no drug/dose changes in the prior 2 weeks, New York Heart Association (NYHA) class I to III symptoms, no contraindications to magnetic resonance imaging (MRI), no use of erythropoiesis‐stimulating agents, immunosuppressive agents or chemotherapy in the prior 3 months, and no active infection, inflammation, renal replacement, or severe chronic obstructive pulmonary disease. Medical and drug histories were obtained from patients and their medical records. A ferritin <100 µg/L or 100–300 µg/L with a transferrin saturation (TSAT) <20% defined ID. A Hb <120 g/L in females and <130 g/L in males defined anaemia. Healthy age‐ and gender‐similar controls with no known medical or drug history were also recruited. The local research ethics committee and institutional review boards approved the study which complied with the International Conference on Harmonization for Good Clinical Practice and the Declaration of Helsinki. All subjects gave written informed consent prior to inclusion.

### Cardiac ^31^phosphorus‐magnetic resonance spectroscopy

**Figure 1:**
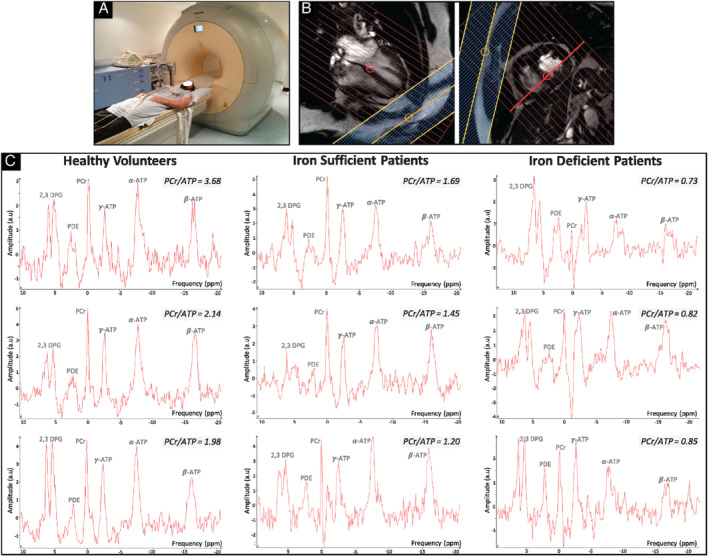
Cardiac ^31^phosphorus magnetic resonance spectroscopy (^31^P‐MRS). Subjects are positioned supine with the ^31^P coil overlying the heart (*A*). Participants are then moved head‐first into the scanner. Pre‐saturation bands (blue grid with yellow border) are applied across the chest wall to prevent spectral contamination from skeletal muscle, and the voxel encompassing the bulk of the interventricular septum was used for quantification of phosphocreatine (PCr) and adenosine triphospahte (ATP) resonances (*B*). Example spectra from three subjects per study group are shown (*C*).

**Figure 2:**
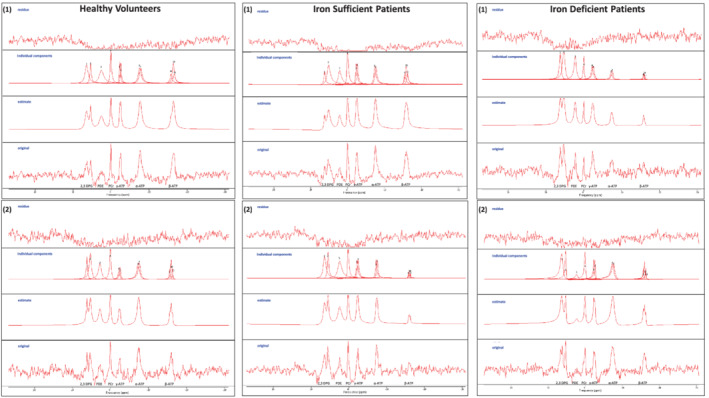
Cardiac ^31^phosphorus magnetic resonance spectroscopy spectral fitting. Representative results from two subjects per study group of the fitting procedure using AMARES with prior knowledge are shown. For each subject, the original spectrum (bottom), individual peaks fitting (second from top), final spectral fit (second from bottom), and residual (top) is depicted.

Cardiac ^31^P‐MRS was performed on a 3 Tesla Achieva MR scanner using a 14 cm diameter linearly polarized transmit‐receive ^31^P surface coil (both from Philips, Best, The Netherlands). Participants were positioned supine (*Figure* *1A*) and moved head‐first into the scanner until their heart was at magnet isocenter. ^1^H‐MR scout images confirmed placement of the ^31^P coil over the interventricular septum. After B_0_ shimming, a slice‐selective, cardiac‐gated one‐dimensional chemical shift imaging sequence was used with presaturation bands applied across the chest wall to avoid spectral contamination from skeletal muscles. Sixteen coronal phase‐encoding steps were used, yielding spectra from 10‐mm slices (temporal resolution = heart rate, time delay = 400 ms, flip angle = 50°), with a total acquisition time of ∼25 min. Spectral locations were overlaid onto an anatomical image and the slice containing the interventricular septum was used for quantification (*Figure* *1B* and *C*). Spectral analysis was performed offline using jMRUI v5.2 and applying the AMARES algorithm with prior knowledge (*Figure* *2*).^18^ The 2,3‐diphosphoglycerate (2,3‐DPG), PCr, γ‐ATP, α‐ATP, and β‐ATP peaks were quantified. The PCr/ATP ratio reported is the blood‐ and saturation‐corrected PCr/γ‐ATP ratio as previously described.^19^ To improve data precision, multiple analyses of each spectrum were performed and the average of the replicates used for comparisons.

### Cardiac magnetic resonance imaging

After ^31^P spectra acquisition, a stack of short‐axis balanced steady‐state free precession images covering the left ventricle was acquired during breath‐holds for assessment of cardiac morphology and function. This was analysed offline as previously described.^19^

### Statistics

Data are presented as mean ± standard deviation, median [interquartile range], or frequencies (%). Normality of distribution was assessed using the Shapiro–Wilks test. Continuous variables were compared using a Student's *t*‐test, Mann–Whitney U test, ANOVA with Bonferroni correction, or a Kruskal–Wallis test with Bonferroni correction. Adjusted analyses were done with an ANCOVA using log‐transformed variables if appropriate. Categorical data were compared using Pearson's χ^2^ test or Fisher's exact test. Pearson's and Spearman rank coefficients were determined to assess correlations. A sample size of 26 patients in total was required to detect a difference of 0.5 in the PCr/ATP ratio^20^ between patients with and without ID at 80% power at an alpha of 0.05 and assuming a standard deviation of 0.45. A two‐tailed *p*‐value <0.05 was considered significant. All statistical analyses were performed using R version 3.6.2 and SPSS version 27 (IBM SPSS, New York, NY, USA).

## Results

### Baseline characteristics

Of 11 healthy volunteers, 12 patients without ID, and 27 patients with ID who were recruited, one patient with ID did not tolerate the MRI, two patients with ID did not have a discernible PCr peak on their spectra, and two patients with ID had poor quality spectra that was presumed to be due to obesity. The baseline characteristics of the study groups were broadly similar (*Table* *1*). All healthy volunteers were Caucasian compared to ∼60% of CHF patients. Compared to patients without ID, those with ID were less likely to be in sinus rhythm (all atrial fibrillation), had greater symptoms as reflected by a higher NYHA class, and, as expected, had lower ferritin and TSAT levels. Only one patient (who had ID) received intravenous iron in the 2 months prior to study recruitment, and no patient received intravenous iron after study recruitment and before MRS examinations.

**Table 1:** Baseline characteristics

	Healthy volunteers (*n* = 11)	Patients with CHF		*p*‐value
		Iron‐sufficient (*n* = 12)	Iron‐deficient (*n* = 27)	
Age, years	60 ± 8	68 ± 10	68 ± 10	0.08
Male sex	5 (45)	10 (83)	14 (52)	0.12
Caucasian ethnicity	11 (100)	7 (58)	17 (63)	0.04
LVEF, %	–	40 [32–45]	35 [26–43]	0.15
NYHA class, %				<0.001
I	–	7 (58)	0 (0)	
II or III	–	5 (42)	27 (100)	
Body mass index, kg/m^2^	25 ± 3	27 ± 5	29 ± 6	0.21
Coronary artery disease	–	0 (0)	2 (7)	0.33
Hypertension	–	9 (75)	16 (59)	0.34
Diabetes	–	4 (33)	8 (30)	0.82
Sinus rhythm	11 (100)	12 (100)	19 (70)	0.03
Heart rate, bpm	68 ± 14	61 ± 7	70 ± 14	0.18
Systolic BP, mmHg	–	132 (17)	128 (18)	0.51
Diastolic BP, mmHg	–	72 (12)	68 (14)	0.41
Ferritin, µg/L	–	163 [143–294]	67 [48–83]	<0.001
Transferrin saturation, %	–	27 [24–37]	17 [14–23]	<0.01
Haemoglobin, g/L	–	137 [127–147]	133 [118–141]	0.29
eGFR, ml/min/1.73 m^2^	–	71 [59–86]	69 [48–76]	0.19
Medications				
ACE‐I/ARB/ARNI	–	12 (100)	25 (93)	0.54
β‐blockers	–	12 (100)	23 (85)	0.16
MRA	–	7 (58)	12 (44)	0.42
Diuretics	–	5 (42)	14 (52)	0.56
Digoxin	–	0 (0)	1 (4)	0.50

Data are mean ± standard deviation, median [interquartile range], or frequency (*n*, %). *p*‐values refer to two or three group comparisons with appropriate *post‐hoc* testing.

ACE‐I, angiotensin‐converting enzyme inhibitor; ARB, angiotensin receptor blocker; ARNI, angiotensin receptor–neprilysin inhibitor; BP, blood pressure; CHF, chronic heart failure; eGFR, estimated glomerular filtration rate; LVEF, left ventricular ejection fraction; MRA, mineralocorticoid receptor antagonist; eGFR, estimated glomerular filtration rate (according to the Modification of Diet in Renal Disease equation); NYHA, New York Heart Association.

### Cardiac energetics

Representative ^31^P‐MRS spectra are shown in *Figure* *2*. 2,3‐DPG relative signal intensities were similar (*p* = 0.99) in healthy volunteers (0.28 [0.22–0.34]) and patients without (0.28 [0.24–0.34]) and with (0.28 [0.22–0.34]) ID. This suggests that the degree of spectral blood contamination was comparable between groups. The PCr relative signal intensity progressively declined (*p* = 0.05) from healthy volunteers (0.47 ± 0.30) to patients without ID (0.39 ± 0.18), to patients with ID (0.29 ± 0.14). The γ‐ATP relative signal intensity was similar (*p* = 0.92) in healthy volunteers (0.20 [0.16–0.30]), and patients without (0.22 [0.15–0.28]) and with (0.22 [0.11–0.32]) ID. Calculated total ATP relative signal intensities progressively declined (*p* = 0.10) from healthy volunteers (0.33 [0.29–0.39]), to patients without (0.26 [0.16–0.30]) and with (0.20 [0.13–0.40]) ID.

**Figure 3:**
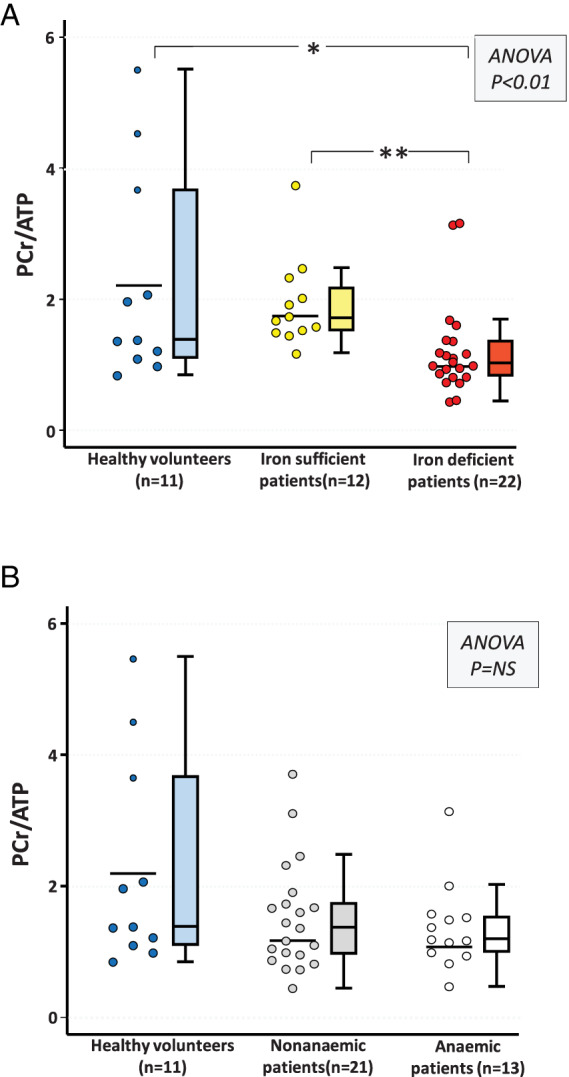
Phosphocreatine to adenosine triphospahte (PCr/ATP) ratio in healthy volunteers and chronic heart failure patients stratified for iron (*A*) and anaemia (*B*) status. For individual datapoints, the solid line depicts the mean. For box‐and‐whisker plots, whiskers represent 90th and 10th percentiles, upper and lower end of boxes represent 75th and 25th percentiles, and solid line depicts median. *p < 0.05, **p < 0.01 (Kruskal–Wallis post‐hoc testing with Bonferroni correction).

**Figure 4:**
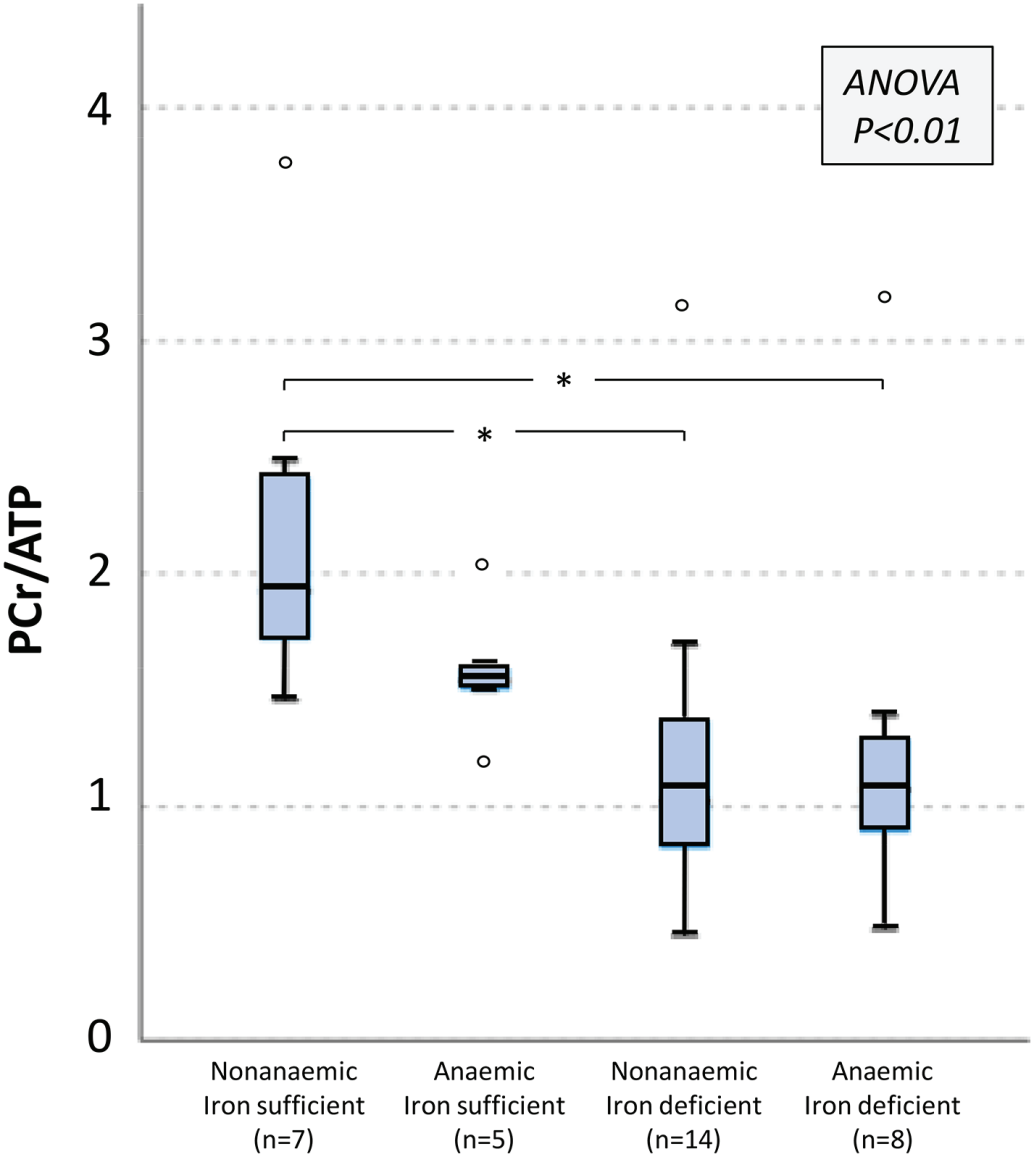
Phosphocreatine to adenosine triphospahte (PCr/ATP) ratio in chronic heart failure patients stratified by anaemia and iron status. In box‐and‐whisker plots, whiskers represent 90th and 10th percentiles, upper and lower end of boxes represent 75th and 25th percentiles, and solid line depicts median. *p < 0.05 (Kruskal–Wallis post‐hoc testing with Bonferroni correction).

The PCr/ATP ratio significantly differed between the study groups (*p* = 0.001, *Figure* *3A*) with patients with ID having a lower PCr/ATP ratio than patients without ID (1.03 [0.83–1.38] vs 1.72 [1.51–2.26], *p* = 0.002) and healthy volunteers (1.39 [1.10–3.68], *p* = 0.04). In contrast, the PCr/ATP ratio did not significantly differ between patients without ID and healthy volunteers. The PCr/ATP ratio also did not differ when patients were stratified on the basis of anaemia (*Figure* *3B*). Categorization on the basis of both anaemia and ID (*Figure* *4*) revealed a progressive decline in the PCr/ATP ratio from non‐anaemic patients without ID to anaemic patients with ID, to non‐anaemic patients with ID, to anaemic patients with ID. The PCr/ATP ratio did not differ between anaemic and non‐anaemic patients with ID. In all CHF patients, higher PCr/ATP ratios correlated modestly with higher ferritin levels (rho = 0.4, *p* = 0.01), but not with Hb or TSAT levels. Stratification on the basis of NYHA class revealed that the PCr/ATP ratio was higher in class I (*n* = 7, 1.93 [1.59–2.48]) than class II (*n* = 20, 1.13 [0.84–1.66], *p* = 0.02) or III (*n* = 7, 1.18 [1.00–1.39], *p* = 0.05) patients (ANOVA *p* = 0.02). In a *post‐hoc* sensitivity analysis, inclusion of the two patients with suboptimal ^31^P‐MRS spectra did not alter the results.

To assess whether the observed variance in the PCr/ATP ratio was due to Hb levels, the presence of anaemia or diabetes, or to intergroup differences in baseline characteristics, separate ANCOVA models were constructed. The PCr/ATP ratio remained significantly different between patients with and without ID after univariable adjustment for gender (adjusted *post‐hoc p* = 0.005), ethnicity (adjusted *post‐hoc p* = 0.005), Hb levels (adjusted *post‐hoc p* = 0.005), presence of anaemia (adjusted *post‐hoc p* = 0.002), presence of diabetes (adjusted *post‐hoc p* = 0.009), heart rhythm (adjusted *post‐hoc p* = 0.001), NYHA class (adjusted *post‐hoc p* = 0.02), and use of β‐blockers (adjusted *post‐hoc p* = 0.004).

### Cardiac structure and function

**Figure 5:**
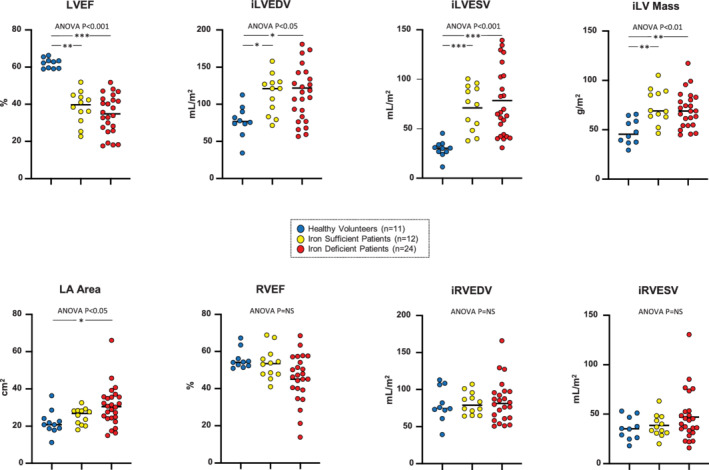
Cardiac structure and function. Magnetic resonance imaging parameters in the study groups. Individual datapoints are shown and the solid line depicts the mean. Indexed values are given and are denoted by the prefix i. Data were available for 24 iron‐deficient patients as one patient did not tolerate magnetic resonance imaging and two had insufficient/inadequate images. *p < 0.05, **p < 0.01. iLV, indexed left ventricular; iLVEDV, indexed left ventricular end‐diastolic volume; iLVESV, indexed left ventricular end‐systolic volume; iRVEDV, indexed right ventricular end‐diastolic volume; iRVESV, indexed right ventricular end‐systolic volume; LA, left atrial; LVEF, left ventricular ejection fraction; RVEF, right ventricular ejection fraction.

Despite a lower PCr/ATP ratio in CHF patients with ID compared to those without, there was no significant difference in measured indices of cardiac structure and function between these groups (*Figure* *5*). Although not significant, patients with ID had numerically lower LVEFs, higher indexed left ventricular end‐systolic volumes, higher left atrial areas, lower right ventricular (RV) ejection fractions, and higher indexed RV end‐systolic volumes than patients without ID. Only one patient (who did not have ID) had septal flattening in diastole which is a surrogate for RV pressure overload. As expected, left ventricular indices were significantly worse in CHF patients than in healthy volunteers, but RV indices did not differ between these groups. To further confirm that differences in the PCr/ATP ratio were independent of the degree of adverse ventricular remodelling, an ANCOVA adjusting for LVEF was done. Differences in energetics between patients with and without ID persisted (adjusted *post‐hoc p* = 0.003) after this adjustment.

## Discussion

Iron deficiency is prevalent in CHF and might escalate symptoms and exercise intolerance by suppressing cardiac energetics. Despite extensive animal data suggesting that ID impairs cardiomyocyte energy generation, only one study has attempted to corroborate this in humans.^15^ In that study, *ex‐vivo* mitochondrial respiration was assayed using cardiac homogenates. Here, we utilised ^31^P‐MRS which is the gold‐standard non‐invasive means of quantifying myocardial bioenergetics *in vivo*.

To the best of our knowledge, this is the first study to show that cardiac energetics, as reflected by the PCr/ATP ratio on ^31^P‐MRS, is diminished in CHF patients with ID compared to those without. This was evident despite no intergroup difference in cardiac structure and function, and persisted after adjustment for the presence of anaemia. The PCr/ATP ratio was also lower in iron‐deficient patients compared to healthy volunteers, but did not significantly differ between iron‐sufficient patients and healthy volunteers. In the total CHF cohort, lower PCr/ATP ratios related to lower ferritin levels, and to greater symptoms and disease severity as reflected by a higher NYHA class.

Cardiac energetics, as measured *ex vivo* using high resolution respirometry, was preserved in a prior study in iron‐deficient CHF patients.^15^ This might be because cardiac homogenates were used which yield lower maximal respiratory capacities and more uncoupled mitochondria than permeabilised muscle fibres.^21^ More importantly, *in‐vivo* mitochondrial assessments (e.g. with ^31^P‐MRS) are, in principle, better than *ex‐vivo* assessments. Due to variability in the acquisition and analysis of published PCr/ATP ratios, it is difficult to compare across studies but our ratios were within the range of values reported for healthy subjects and CHF patients,^16^, ^17^, ^22^, ^23^, ^24^, ^25^ implying that they were likely physiologically relevant.

Iron is quantitatively the most important biocatalyst in oxidative metabolism, so greater cardiac energetic impairment with ID is biologically plausible. Of the ATP needed by the heart to function optimally, OXPHOS provides over 90%. Iron is implicated at every step of OXPHOS. It functions in Krebs cycle and fatty acid β‐oxidation enzymes, in complex I to IV of the ETC, in Hb and myoglobin, and in catalase which limits redox inactivation of ATP synthase and creatine kinase.^6^, ^7^, ^8^, ^9^, ^10^ Accordingly, rodent hearts with ID are consistently energetically compromised and we now forward this observation to humans.^12^, ^13^ Besides attenuating ATP supply, ID might also lower the PCr/ATP ratio via other mechanisms.

Left ventricular hypertrophy, dilatation, and dysfunction due to ID could enhance myocyte ATP demand to reduce the PCr/ATP ratio.^13^, ^26^ However, as cardiac structure and function did not significantly differ between our CHF groups, the link between ID and reduced PCr/ATP ratios in CHF is likely mediated via reductions in ATP supply. That cardiac structure and function did not differ despite attenuated energetics with ID might be due to our matching of patients for variables collinear with LVEF, to inadequate statistical power, or to ID exerting a far greater effect on myocardial energetics than on left ventricular remodelling in CHF.

Poorer cardiac energetics was observed in patients with a higher NYHA class, implying that cardiac mitochondrial dysfunction due to ID could be a mechanism for dyspnoea, exercise intolerance, and disease progression in CHF, and that improved cardiac energetics may be a route via which intravenous iron repletion is beneficial.^3^ That patients with ID had greater energetic impairments even after adjustment for NYHA class suggests that ID might directly suppress mitochondria in addition to indirectly doing so by worsening the syndrome.

Several other findings deserve mention. First, anaemia did not correlate with the PCr/ATP ratio likely due to its myriad causes in CHF that may not all similarly impact energetics. Moreover, ID was linked with the PCr/ATP ratio irrespective of anaemia, in line with CHF data showing that ID is ominous independently of anaemia,^1^ that iron is beneficial irrespective of Hb levels,^3^, ^27^ and that oxygen delivery does not limit muscle metabolism.^28^ Second, the PCr/ATP ratio correlated with ferritin but not with TSAT possibly due to our criteria for ID which has a broader range for ferritin. Skeletal muscle metabolic changes during exercise also only correlated with serum ferritin levels in a prior CHF study.^29^ Third, the PCr/ATP ratio did not differ between iron‐sufficient patients and healthy subjects, tempting the speculation that ID might be the dominant cause of myocardial energy deficiency in CHF. This similarity might be because our controls had relatively low ratios that were still within the published range in healthy subjects,^16^, ^17^, ^22^, ^23^, ^24^, ^25^ and/or because we only quantified the ratio in unstressed hearts. In line with our data, Melenovsky *et al*.^29^ also found no difference in key skeletal muscle energetic indices between healthy controls and patients without ID. In contrast, they found similar skeletal muscle energetics (as quantified using PCr recovery kinetics) in CHF patients who did and did not have ID. This disparity with our data may reflect a greater sensitivity of the heart to energetic insults from ID given the hearts higher energetic requirements compared to skeletal muscle.^23^ The disparity may also reflect the relatively high exercise loads used to assess skeletal muscle PCr recovery^29^ which can evoke anaerobic metabolism to confound the assessment of OXPHOS.

Our study's strength is the use of ^31^P‐MRS, but it also has limitations. First, it is observational so causality cannot be inferred. Second, we were likely underpowered for many secondary analyses, but were adequately powered for the primary comparison which was that of the PCr/ATP ratio between patients with and without ID. Third, patients with ID were more symptomatic and less likely to be in sinus rhythm than those without ID, but the difference in cardiac energetics between these groups persisted even after ANCOVA adjustment for NYHA class, cardiac rhythm, and other potential confounders such as Hb levels, anaemia, and diabetes. Fourth, we did not scan patients at 1.5 Tesla which would have enabled optimal quantification of cardiac iron content (T2*) to inform this study. Fifth, iron status and Hb were not measured in healthy volunteers, but their absence does not detract from our main findings. Sixth, skeletal (intercostal) muscle contamination of cardiac ^31^P‐MRS signals can occur but the discordance between our results and those obtained in skeletal muscle^29^ suggests that this cannot account for our findings. For example, compared to the skeletal muscle data, we found greater progressive reductions in resting PCr (38% vs. 2%) and total ATP (39% vs. 11%) signals from controls to patients with ID, and significantly lower resting energetic ratios (PCr/ATP) with ID whereas the ratios (PCr/Pi, ATP/Pi) did not differ in skeletal muscle.

Our study has clinical ramifications. First, it reinforces the importance of clinicians actively seeking, aggressively investigating, and promptly correcting, systemic ID in CHF which has been shown to augment myocardial iron content.^30^ Second, it suggests that resting cardiac structure and function may not reliably gauge the impact of ID on the heart.

In conclusion, we show for the first time that cardiac energetics, as reflected by the PCr/ATP ratio on ^31^P‐MRS, is diminished in CHF patients with ID compared to those without. This was evident despite no intergroup difference in cardiac structure and function, and persisted after adjustment for anaemia. Lower PCr/ATP ratios related to greater symptoms and disease severity as reflected by a higher NYHA class in CHF patients.
